# Retraction: A deep facial recognition system using computational intelligent algorithms

**DOI:** 10.1371/journal.pone.0342725

**Published:** 2026-02-12

**Authors:** The PLOS One Editors

The *PLOS One* Editors retract this article [1] due to concerns about:

- The use of terminology that differs from standards in the field (e.g., ‘enormous information’ instead of ‘big data’).
- The subsection 4.2 Fundamentals of transfer learning in section 4. Proposed facial recognition system appears similar in content to a previously-published article [2] that was not cited.
- Compliance with PLOS policies on Data Availability.
- Inconsistencies within the article regarding the source of datasets used in the study.

All authors either did not respond directly or could not be reached.
